# Chinese Medicines in the Treatment of Prostate Cancer: From Formulas to Extracts and Compounds

**DOI:** 10.3390/nu10030283

**Published:** 2018-02-28

**Authors:** Xueni Wang, Gang Fang, Yuzhou Pang

**Affiliations:** 1 Guangxi Zhuang Yao Medicine Center of Engineering and Technology, Guangxi University of Chinese Medicine, 13 Wuhe Road, Qingxiu District, Nanning 530200, China; 000831@gxtcmu.edu.cn (X.W.); 000387@gxtcmu.edu.cn (Y.P.); 2 Laboratory of Zhuang Medicine Prescriptions Basis and Application Research, Guangxi University of Chinese Medicine, 179 Mingxiudong Road, Xixiangtang District, Nanning 530001, China

**Keywords:** prostate cancer, Chinese medicines, formulas, extracts, compounds

## Abstract

In order to fully understand the progresses and achievements in Chinese medicines for the treatment of prostate cancer, we summarize all the available reports on formulas, extracts, and compounds of Chinese medicines against prostate cancer. A number of clinical trials verified that traditional Chinese formulas had some unique advantages in the treatment of prostate cancer. Many Chinese medicine extracts could protect against prostate cancer, and many compounds isolated from Chinese traditional medicines showed a clear anti-prostate cancer effect. However, Chinese medicines are facing many problems regarding their multicomponent nature, complicated mechanisms of action, and high doses required for therapy. Herein, we review the functions of Chinese medicines in prostate cancer and focus on their mechanisms. The review will deepen the understanding of Chinese medicines potential in the anti-prostate cancer field. In addition, we put forward a question concerning the current research on Chinese medicines: in order to better illustrate that Chinese medicines can be used in the clinical treatment of prostate cancer, should our research focus on formulas, extracts, or compounds?

## 1. Introduction

Prostate cancer (PCa) is a common cancer in elderly men. The incidence of PCa is different in different countries, and the general mortality rate of prostate cancer reaches 1–2% [1]. The mechanisms of PCa progression have been explored, and key progresses have been made in the treatment of PCa. Personalized therapies based on nanomedicines, Chinese medicines, and genetic factors, have become one of the study hotspots of PCa treatment [2]. However, why are the complex Chinese medicines increasingly concerned?

Because of their low toxicity, non-apparent side effects, and obvious curative effects in tumor treatment, Chinese medicines have been accepted by doctors, experts, and patients [3]. Chinese medicines can inhibit tumor growth and metastasis, prolong patients’ life span, and improve patients’ life quality [4]. Some patients, who were found to have cancers in the late stage, could not undergo surgery, radiotherapy, and chemotherapy, and finally accepted Chinese medicines treatment, which restrained the tumor progression, improved their quality of life, and obviously extended their survival [4]. Herbal medicines are the traditional medicine resources in China, with unique advantages. Medicinal plant resources are widely distributed in China. More than 50% of anticancer drugs approved by the U.S. Food and Drug Administration (FDA) are extracted from terrestrial plants [5], indicating that herbal medicines are important in the development of anticancer drugs.

Traditional Chinese medicine formulas are widely used in clinical cancer treatment in Chinese hospitals [6]. Many Chinese herbal extracts have been proved to inhibit the development of prostate cancer. More and more compounds isolated from Chinese medicine herbs were found to inhibit the development of prostate cancer via different pathways. A number of clinical trials have shown that traditional Chinese medicines play a definite role in the protection against prostate cancer and will be used in clinical treatment of prostate cancer in the future. Chinese medicines have a great potential in PCa treatment.

Pharmacological research on the antitumor efficacy of Chinese herbal extracts and formulas is developing promptly, and the application of Chinese medicine compounds in clinical oncology has been widely accepted. Soiqnet et al. established arsenic trioxide as an effective therapy for patients with relapsed acute promyelocytic leukemia and recorded high rates of 5-year disease-free survival and 5-year overall survival after a long-term follow-up of newly diagnosed patients with acute promyelocytic leukemia treated with a single-agent therapy based on arsenic trioxide [6,7]. The combination of Chinese medicines and other treatment methods has been widely used in China and has achieved a good therapeutic efficacy [8,9,10,11]. Although studies on Chinese medicines in anticancer therapy have demonstrated some achievements, there is still a long way for the wide application of Chinese medicines in the treatment of prostate cancer. This work intends to summarize the recent progresses in the use of Chinese medicines for PCa treatment and points out possible future breakthroughs.

## 2. Chinese Medicine Formulas in the Treatment of Prostate Cancer

PC-SPES is a Chinese medicine formula containing eight herbal medicines. It was launched into the market as a health care product in 1996 by Botanic Lab [12]. The New England Journal of Medicine first reported the biological activity and the clinical effect of PC-SPES in 1998 [13]. A series of reports showed that PC-SPES can significantly reduce serum testosterone and prostate-specific antigen in patients with prostate cancer in a time- and dose-dependent manner [14,15,16,17]. However, in 2001, patients taking PC-SPES showed diffuse intravascular coagulation and bleeding tendency [18,19]. In 2002, some batches of PC-SPES were found to contain diethylstilbestrol, warfarin, and indomethacin, which made the efficacy and side effects of PC-SPES controversial [20]. Considering the stability and safety of Chinese herbal remedies, National Center for Complementary and Alternative Medicine (NCCAM) suspended research on PC-SPES and its related compounds and withdrew it from the USA market in February 2000 [21].

From 2010 to 2012, Lin et al. reported a series of clinical data on prostate cancer treated with Chinese medicines. Firstly, they found that traditional Chinese medicine (TCM) treatment was only a complementary treatment rather than an alternative treatment. Then, they found that TCM treatment showed an increasing trend in popularity among prostate cancer patients in Taiwan. Later, they confirmed that TCM treatment was popular among prostate cancer patients in Taiwan [22,23,24,25].

A retrospective nationwide cohort study of prostate cancer patients was conducted based on data from 1998 to 2003 from the Taiwan National Health Insurance Research Database. Patients were classified as TCM users or nonusers and monitored from the day of prostate cancer diagnosis to death or to the end of 2012. The correlation between death risk and TCM use was determined with cox-proportional-hazards models and Kaplan–Meier curves. The correlation analysis results showed TCM users had a decreased mortality. A lower death risk was observed in patients with longer-term TCM use, especially in those who used TCM for at least 200 days. TCM users with metastatic prostate cancer had a significantly lower hazard ratio than non-TCM users [26].

PC-SPES’s entry into the US market has convinced that this Chinese medicine formula has a definite efficacy on prostate cancer, although PC-SPES was withdrawn from the US market after 4 years. Chinese medicine researchers realize that it is a key issue to ensure Chinese medicines’ stability and safety. Many scientists in China today are devoted to the quality control of Chinese medicines.

A leading cause of the prostate cancer treatment failure is chemoresistance, which often involves multiple mechanisms. Chinese medicines usually contain multiple components, which can potentially target many mechanisms simultaneously and may offer an advantage over single compounds. The characteristic of Chinese medicines can be used as a strategy to avoid chemoresistance.

Chinese medicines have been applied for a long time. The mechanisms of Chinese medicines should be further explored with advanced scientific and technological methods to control the stability and safety of Chinese medicines for human health.

## 3. Chinese Medicine Extracts Inhibit Prostate Cancer

Traditional plant-derived products play a significant role in the development of potential medicinal agents. *Ganoderma lucidum* (Leyss. ex Fr.) Karst. is a type of mushroom and has been used as a home remedy for the general promotion of health and longevity in traditional Chinese medicines [27]. Spores and unpurified fruiting bodies of *G. lucidum* (0.5–2.5 mg/mL) could inhibit the invasion of breast MDA-MB-231 and prostate PC-3 cancer cells by downregulating the expression of NF-kappaB, urokinase plasminogen activator (uPA), and uPA receptor [28]. Meanwhile, *G. lucidum* (0.125–0.5 mg/mL) could induce apoptosis, inhibit cell proliferation, and suppress the migration of highly invasive PC-3 human prostate cancer cells [29]. Furthermore, *G. lucidum* (0.125–0.5 mg/mL) was reported to inhibit prostate cancer-dependent angiogenesis by modulating MAPK and Akt signaling in PC-3 cells [30]. These results indicated that *G. lucidum* had a potential therapeutic efficacy for the treatment of prostate cancer.

*Litchi chinensis* Sonn. (Litchi) is a subtropical fruit tree growing in south China. Litchi seed extracts were found to possess diverse pharmacological effects including significantly inhibiting cell viability and clonogenic growth of prostate cancer PC-3, DU145, RM-1, and C4-2B cells in a dose-dependent manner (31.25–250 μg/mL) and inducing cell apoptosis and cell cycle G1/S phase arrest by inactivating protein kinase B (Akt or PKB) signaling pathway [31]. In addition, the extracts significantly decreased cell migration and invasion via a phenotypic inversion of epithelial–mesenchymal transition [31]. Remarkably, the extracts significantly decreased the size of PC3 xenograft nude mice, showing no toxicity [31]. These findings suggested that Litchi seed extracts might be used to develop a safe alternative therapy for prostate cancer patients.

*Saussurea lappa* Clarke has been applied in the treatment of inflammation, abdominal pain, tenesmus, nausea, and cancer in China [32,33]. Hexane extracts of *S. lappa* inhibited the basal and Epidermal Growth Factor (EGF)-induced migration of prostate cancer DU145 and TRAMP-C2 cells in a dose-dependent manner (1–4 μg/mL), whereas they did not influence the viability of these cancer cells. In addition, the extracts reduced matrix metalloproteinase (MMP)-9 and tissue inhibitor of metalloproteinase (TIMP)-1 secretion, but increased TIMP-2 levels in the absence or presence of EGF [34]. The results indicated that hexane extracts of *S. lappa* might be used as anti-metastatic agents for the treatment of prostate cancer.

*Scutellaria baicalensis* Georgi is a widely used Chinese herbal medicine in anti-inflammatory and anti-cancer therapy [35,36,37]. *S. baicalensis* (0.2–0.8 mg/mL) exerted dose- and time-dependent growth inhibition effects on both LNCaP and PC3 cell lines and also inhibited prostate-specific antigen production in LNCaP cells. Animal experiments of *S. baicalensis* showed a reduction of 50% in tumor volume after a 7-week treatment at a dose of 200 mg/kg/day [38]. These results imply that *S. baicalensis* possess anti-prostate cancer activity in vitro and in vivo.

*Scutellaria barbata* D. Don has been used to treat various cancers in China [39,40,41]. Wong et al.’s in vivo data showed that *S. barbata* (32 mg/day) delayed tumor development in a transgenic prostate adenocarcinoma mouse model ,and the complementary in vitro data indicated that *S. barbata* (1 mg/mL) might exert this function by upregulating the apoptotic pathway and downregulating the survival pathway in TRAMP-C1 and LNCaP prostate cancer cells [42]. According to these results, *S. barbata* might possess chemopreventive properties for cancer treatment.

*Tripterygium wilfordii* Hook F (12.5–50 μg/mL) combined with docetaxel could overcome the chemoresistance and suppress prostate tumor growth in docetaxel-resistant PC3 and DU145 prostate cancer cell lines by inhibiting P-glycoprotein activity and inducing a significant change in the expression of genes related to angiogenesis, cell cycle regulation, and differentiation [43]. This findings imply that *T. wilfordii* might be developed as a combined agent to prevent chemoresistance.

*Wedelia chinensis* (Osbeck) Merr. is a common ingredient in anti-inflammatory herbal medicines in China [44,45,46]. The anti-prostate cancer effect of *W. chinensis* extracts is ascribed to three active compounds: wedelolactone, luteolin, and apigenin, which inhibit the androgen receptor (AR) signaling pathway in LNCaP and 22Rv1 cells. Oral administration of *W. chinensis* extracts (4 or 40 mg/kg) impeded prostate cancer tumorigenesis [47].

On the basis of the above-mentioned results, we found that these Chinese medicines had anti-prostate cancer activity (Table 1). These results also suggested that many potential natural compounds with anti-prostate cancer activity might exist in Chinese herbal extracts.

## 4. Compounds Isolated from Chinese Medicines Inhibit Prostate Cancer

### 4.1. Compounds Inhibiting the Growth of Prostate Cancer Cells

Andrographolide is a diterpenoid lactone derived from a traditional Chinese medicine plant *Andrographis paniculata* (Burm. f.) Nees. Chun et al. demonstrated that andrographolide (1–20 μM) could inhibit IL-6-induced signaling and induce signal transducer and activator of transcription 3 (STAT3) and the phosphorylation of extracellular signal-regulated kinases (ERK). Meanwhile, it could inhibit cell viability and induce apoptosis of PC3 and DU145 prostate cancer cell lines. Moreover, andrographolide suppressed tumor growth in a mouse xenograft model with castration-resistant DU145 cell-derived human prostate tumors. These findings imply that andrographolide could be developed as a therapeutic agent to treat prostate cancer [48].

Evodiamine is isolated from the Chinese herbal medicine *Evodia rutaecarpa* (Juss.) Benth. Recent results showed that evodiamine suppressed the growth of LNCaP cells in a dose-dependent manner (100 nM–100 μM) by arresting the cell cycle at the G2/M phase and inducing apoptosis [49].

Guttiferone F (10 μM and 20 μM) could induce apoptosis of LNCaP and PC3 prostate cancer cells under serum starvation via JNK activation and Ca^2+^ elevation, respectively. Furthermore, it exerted a significant growth inhibition on PC3 cells xenografts in vivo at a dose of 20 mg/kg [50].

Honokiol is a compound contained in the traditional Chinese herb *Magnolia officinalis* Rehd. et Wils. The viability of PC-3 and LNCaP human prostate cancer cells was decreased when the cells were treated with honokiol (20–60 μM). These results were confirmed to be correlated with G0/G1 phase cell cycle arrest [51]. Honokiol (5–20 μM) induced apoptosis in PCa cell lines by activating caspases 3/8/9 and cleaving poly-adenosine diphosphate ribose polymerase. In addition, honokiol (100 mg/kg) combined with docetaxel (5 mg/kg) showed growth-inhibitory, apoptotic, and antiangiogenic effects in C4-2B tumor xenografts [52]. Moreover, the exposure of PC-3, LNCaP, and Myc-CaP cells to 20–40 μM honokiol resulted in ROS-mediated cytoprotective autophagy [53].

Isorhapontigenin (ISO) is widely distributed in Chinese herbs, fruits, and vegetables [54,55,56]. Isorhapontigenin (ISO, 20–100 μM) induced cell growth inhibition and apoptosis of LNCaP and 22Rv1 by targeting EGFR and its downstream signal pathways and showed no obvious effects on normal prostate cells [57]. Moreover, the treatment with ISO decreased the protein level of AR by promoting the ubiquitination and degradation of AR proteins in proteasome and inhibiting the expression of AR gene [57]. In vivo, 50 mg/kg of ISO inhibited the growth of subcutaneously xenotransplanted tumors in nude mice by inducing PCa cell growth inhibition and apoptosis [57]. EGFR-related signal pathways are involved in ISO-induced cell growth inhibition and apoptosis in PCa cells, suggesting the application potential of ISO in prostate cancer treatment.

Peperotetraphin is a novel cyclobutane-type norlignan isolated from the whole plant of *Peperomia tetraphylla* [58]. Peperotetraphin (50–100 μM) inhibited the growth of PC-3 cells and induced cancer cells to undergo G1 phase arrest and apoptosis [59].

Quercetin is a natural polyphenolic compound widely distributed in Chinese herbal medicines [60]. A large body of evidence showed that quercetin could induce the apoptosis of prostate cancer cells via multiple possible signaling pathways [61,62,63,64,65]. Liu et al. reported that 50–200 μM quercetin might induce apoptosis by direct activation of the caspase cascade via the mitochondrial pathway and endoplasmic reticulum stress in PC-3 cells [66].

Tetrandrine is an alkaloid from the traditional Chinese medicine *Stephania tetrandra* S. Moore [67]. Tetrandrine (2.5–30 μM) inhibited the growth and cell cloning of PC-3 and DU145 cells [68]. In addition, tetrandrine induced apoptosis by inhibiting phosphoinositide 3-kinase–Akt signal pathway and by activating the caspase cascade [68].

Triptolide is one of the main active components of *Tripterygium wilfordii* Hook.f [69]. Tumor necrosis factor-related apoptosis-inducing ligand (TRAIL) is a promising cancer therapy agent [70]. Although a number of prostate cancer cells exhibited high resistance to TRAIL effect [71], 50–200 nM triptolide significantly sensitized LNCaP and PC-3M prostate cancer cells to TRAIL-mediated cellular apoptosis by upregulating the expression of death receptor 5 and inhibiting prostate cancer development [72].

Vitexicarpin is a polymethoxy flavone isolated from *Vitex rotundifolia* Linne fil. and has long been used as an anti-inflammatory herb in China [73]. Meng et al. revealed that vitexicarpin (10–50 μM) induced apoptosis by upregulating the proapoptotic protein Bax, downregulating the antiapoptotic protein Bcl-2, releasing cytochrome C from mitochondria, and decreasing mitochondrial membrane potential in PC3 cells [73].

These Chinese medicine compounds can inhibit the growth of prostate cancer cells mainly by inducing cell apoptosis, arresting the cell cycle at different phases, activating caspase cascade, and/or regulating the expression of apoptosis-related proteins. Moreover, AR signaling pathway and the cytoprotective effect mediated by ROS are also involved in the inhibition effect of Chinese Medicine compounds.

### 4.2. Compounds Inhibiting the Proliferation of Prostate Cancer Cells

Arsenic sulphide is a main component of realgar (As4S4), and 0.5–1 μM arsenic sulphide can repress the overexpression of miRNA-372 in DU145 and PC3 prostate cancer cell lines [74]. An in vivo study confirmed that repressing the overexpression of miR-372 by 2 mg/kg/day of As4S4 for 3 weeks could inhibit the proliferation and migration of prostate cancer cells [74].

Baicalin, isolated from the dried roots of *Scutellaria baicalensis* Georgi, is widely used in China for its anti-inflammatory, anti-pyretic, and anti-hypersensitivity properties [75]. Baicalin (150 μM) could inhibit the proliferation of DU145, PC-3, LNCaP, and CA-HPV-10 prostate cancer cells, and, particularly, of the androgen-independent DU145 cells. It was confirmed that this effect was associated with the induction of apoptosis, although the exact mechanisms are not clear [75].

Pang et al. indicated that celastrol could inhibit prostate cancer development and angiogenesis and that its effects were correlated with the extent of inhibition of AKT/mTOR/P70S6K signaling [76]. Celastrol (0.03–3 μM) could suppress PC-3 cell proliferation by downregulating IL-6 gene expression through the NF-kappaB-dependent pathway [77]. In addition, Yang et al. first reported that celastrol was a natural proteasome inhibitor with a great potential for prostate cancer prevention and treatment [78].

Gypensapogenin H is a novel dammarane-type triterpene from *Gynostemma pentaphyllum* (Thunb.) Makino [79]. Zhang et al. found that 5–60 μM Gypensapogenin H induced apoptosis in DU145 and 22RV-1 human prostate cancer cells by decreasing survival, inhibiting proliferation, and inducing cell cycle arrest in G1 phase [80].

Scoparone, a natural compound isolated from *Artemisia capillaris* Thunb. has anti-allergic effects in a mast cell-mediated allergy model [81]. Kim et al. indicated that scoparone could suppress the transcription of STAT3 and decrease phosphorylation and nuclear accumulation of STAT3 [82]. Scoparone treatment suppressed anchorage-independent growth in soft agar at the concentration of 50–200 μM, and tumor growth of DU145 xenografts in nude mice at the concentration of 30 mg/kg every 2−3 days for 18 days [82]. In addition, computational modeling suggested that scoparone might bind to the SH2 domain of STAT3 [82]. According to these findings, scoparone exerted an anti-prostate cancer effect by inhibiting STAT3 activity.

Triptolide could inhibit the proliferation of RM-1 cells in mice by decreasing the expression of Bcl-2 and increasing the expression of caspase 3 at a dose of 10 and 20 ng/mL [83].

These compounds inhibited the proliferation of prostate cancer cells by repressing the overexpression of miRNA-372, inhibiting inflammation pathways such as the NF-kappaB-dependent pathway, inducing apoptosis, acting as a poroteasome inhibitor, and inhibiting STAT3 activity.

### 4.3. Compounds Inhibiting Metastasis of Prostate Cancer Cells

Metastasis development is still a huge challenge in prostate cancer treatment. Most of the treatment failures of prostate cancer are generally ascribed to the formation of bone metastases [84,85].

Pretreatment with 2–8 μM celastrol significantly slowed down PC3 cell migration [86]. After celastrol administration, the number of cells penetrating a gel layer in a classical invasion assay was significantly decreased, and their bone invasive ability was significantly undermined. Pretreatment with celastrol inhibited the tumorigenicity of PC-3 cells, and almost no bone invasion occurred in an in vivo mouse model [86]. Celastrol could target the VEGFR-2 signaling pathway and inhibit the formation of blood vessel [86].

Curcumin is a well-known natural compound of curcuminoids. Yang et al. found that curcumin could suppress prostate cancer by reducing the function of the PSA promoter and inhibiting PSA and AR protein expression in LNCaP cells [87]. Meanwhile, 10 μM curcumin was demonstrated to inhibit prostate cancer by upregulating miR-143 and FOXD3 and downregulating PGK1 expression in DU145 and PC3 cells [88]. Furthermore, 10–50 μM curcumin could inhibit the metastasis and survival of DU145 and PC3 prostate cancer cells via the Notch-1 signaling pathway [89].

Hu et al. found three novel cyclotides from the leaves and root of *Hedyotis Diffusa* Willd, termed Diffusa cyclotide 1 to 3 (DC1–3). DC3 was found to show potent cytotoxicity against PC3, DU145, and LNCaP prostate cancer cell lines [90]. Furthermore, 0.05 μΜ DC3 inhibited cell migration and invasion of LNCap cells and significantly inhibited tumor development in a prostate xenograft model [90].

D-pinitol inhibited the invasion and migration of PC3 and DU145 prostate cancer cells at the noncytotoxic concentration of 3–30 μM [91]. D-pinitol metastatic activity was mediated by the downregulation of αvβ3 integrin activity through focal adhesion kinase (FAK), c-Src, and NF-κB pathways [91].

Dihydroisotanshinone I, a bioactive compound in *Salvia miltiorrhiza* Bunge. could inhibit the migration of androgen-dependent (22Rv1 cells) and androgen-independent (PC3 and DU145 cells) prostate cancer cells at the concentration of 5 μΜ–10 μΜ [92]. Dihydroisotanshinone I inhibited the migration of these prostate cancer cells by interrupting the cross-talk between macrophages and prostate cancer cells through the repression of the CCL2–STAT3 axis [92].

Shikonin is an active naphthoquinone from the Chinese medicine *Lithospermum erythrorhizon* Sieb. et Zucc. Shikonin potently suppressed PC-3 and DU145 cell metastasis in a dose-dependent manner (0.5–2 μΜ) and inhibited the migration and invasion of the two aggressive prostate cancer cell lines by reducing MMP-2/-9 expression via AKT/mTOR and ROS/ERK1/2 pathways [93].

Transwell invasion assays showed that 2.5–30 μM tetrandrine significantly weakened the invasion capacity of DU145 and PC-3 cells; in addition, this compound exhibited a strong inhibitory effect on the proliferation of these prostate cancer cells [68]. However, the exact molecular mechanisms are still unknown.

Some studies indicated that the tested compounds could inhibit metastasis of prostate cancer cells, but the specific molecular mechanisms are not yet known. Some other findings showed that the tested compounds exerted the inhibitory effect on prostate cancer cell metastasis mainly by inhibiting the VEGFR-2 signaling pathway, regulating the Notch-1 signaling pathway, downregulating FAK, c-Src, and NF-κB pathways, repressing the CCL2–STAT3 axis, and suppressing MMP-2/-9 expression via AKT–mTOR and ROS–ERK1/2 pathways.

### 4.4. Summary

Among the above summarized compounds, Ten compounds showed inhibition of the growth of prostate cancer cells. Six compounds were confirmed to inhibit the proliferation of prostate cells and seven compounds inhibited metastasis of prostate cancer cells. Tetrandrine and celastrol were the only two compounds which could inhibit both growth/proliferation and metastasis of prostate cancer cells. Triptolide suppressed the growth or proliferation of prostate cancer cells was verified in two different studies. Table 2 lists the compounds which can inhibit the growth or proliferation, and metastasis of prostate cancer cells through similar or different targets. These findings suggest that there are numerous natural small molecules which are potentially active against prostate cancer in Chinese medicines. The discovery of these compounds will help to study the anti-prostate cancer activity of Chinese medicines.

## 5. Perspectives and Outlook

In the past years, PCa has been widely studied in China. More and more extracts, formulas, and compounds from traditional Chinese medicines have been found to have anti-prostate cancer activity. Most of these Chinese herbal extracts with anti-prostate cancer activity also show anti-inflammatory activities. Most of the Chinese medicine formulas with anticancer effects have the functions of promoting positive blood flow, removing blood stasis, alleviating fever, and detoxifying. The main compounds present in Chinese medicines with anti-prostate cancer activity are polyphenols, alkaloids, and terpenoids. As shown in Table 3, the extracts of G. *lucidum*, Litchi, S. *lappa* and *T*. *wilfordii* are active in the androgen-independent prostate cancer cells, and S. *barbata* suppresses androgen-dependent prostate cancer cells growth. In addition, the extracts of *S. baicalensis* and *W. chinensis* inhibit both androgen-dependent and androgen-independent prostate cancer cells. Moreover, 8 of the 19 compounds involved in this review, including Guttiferone F, Honokiol, Quercetin, Triptolide, Baicalin, Gypensapogenin H, Curcumin, and dihydroisotanshinone I, inhibit both androgen-dependent and androgen-independent prostate cancer cells. These extracts and/or compounds may be potential anti-prostate cancer agents present in Chinese medicines and deserve further study. The extracts and compounds of Chinese medicines mainly produce the anti-prostate effect in the following ways. Firstly, Chinese medicines induce apoptosis, inhibit cell proliferation, and suppress the migration of invasive human prostate cancer cells. Secondly, Chinese medicines inhibit prostate cancer-dependent angiogenesis and impede prostate cancer tumorigenesis. Thirdly, Chinese medicines induce apoptosis and downregulate survival pathways in prostate cancer cells. Fourthly, Chinese medicines act as anti-metastatic agents. Fifthly, Chinese medicines suppress prostate tumor growth and overcome cell chemoresistance. Chinese herb extracts are a mixture of many compounds at low concentrations, and the effects of the extracts derive from the interactions of all their components. Theompounds isolated from Chinese herbs here discussed perform well in inhibiting the growth, proliferation, and metastasis of prostate cancer cells, and most of them act as selective androgen receptor modulators to exert anti-prostate cancer activity.

In this review, we collected formulas, extracts, and compounds of Chinese medicines with anti-prostate cancer activity and classified their mechanisms of action. We hope these findings can guide and enlighten researchers to explore the use of Chinese medicines in the treatment of prostate cancer.

Chinese researchers have carried out a large number of anti-prostate cancer studies on Chinese medicines under the guidance of the theory of traditional Chinese medicine. Meanwhile, the commonly used research ideas and methods that they have applied are consistent with those used in research for western drugs. The difference is that formulas, extracts, and compounds characteristic of traditional Chinese medicines are natural products. However, traditional Chinese medicines are often not a single Chinese herb extract, but a mixture of many Chinese herbs. Therefore, it is important to note that a Chinese medicine formula is an entirety. Although traditional Chinese medicines have been used for a long time, their mechanism have not been fully elucidated. Therefore, researchers often explored the mechanism of a single compound as a breakthrough point, and then gradually clarified the multi-target and multilevel function of multicomponent Chinese medicines. Chinese scientists and clinicians now are facing unprecedented opportunities and challenges to develop Chinese medicines. In the near future, a research cooperation network for the study of Chinese medicines as anti-prostate cancer agents will be established to impel the use of Chinese medicines in the treatment of prostate cancer.

## 6. Conclusions

This study has reviewed the progresses of Chinese medicines in the field of anti-prostate cancer treatment. Since prostate cancer treatment with Chinese medicines is not popular in the world at present, the available data on their adverse or toxic effects are not sufficient. We did not specifically address the adverse or toxic effects of the formulas, extracts, and compounds discussed in this review. The safety and stability of Chinese medicines have been widely examined, and scientists realize that this is a vacancy to be eliminated. Another point requiring study is the administration dose of Chinese medicines. The human dose of each Chinese medicine involved in a formula was stipulated by the Chinese Pharmacopoeia to ensure safety. The human doses of extracts and compounds should be verified through a series of pharmacological, pharmacokinetic, and toxicological studies. In our research on the bioactivity of Chinese medicines, we found that the administered doses of some extracts or compounds were high. High doses might be decreased by improving the extraction processes or by modifying the structures of the active compounds. There is still a long way to go to discover new anti-prostate cancer agents from Chinese medicines.

## Figures and Tables

**Table 1:** Chinese medicine extracts inhibit prostate cancer.

Scientific Name	Chinese Name	Plant Organs	Extraction Solvents
*Ganoderma lucidum* (Leyss. ex Fr.) Karst.	Ling Zhi	Spores and unpurified fruiting bodies	No solvent
*Litchi chinensis* Sonn.	Li Zhi	Seed	n-butyl alcohol
*Saussurea lappa* Clarke	Guang Mu Xiang	Root	n-Hexane
*Scutellaria baicalensis* Georgi	Huang Qin	Root	Water
*Scutellaria barbata* D. Don	Ban Zhi Lian	Whole plant	Water
*Tripterygium wilfordii* Hook F.	Lei Gong Teng	Root	EtOH
*Wedelia chinensis* (Osbeck) Merr.	Peng Qi Ju	Whole plant	EtOH

**Table 2:** Compounds isolated from Chinese medicines inhibiting prostate cancer.

General Name	Chemical Structure	Compound Classification	Inhibit prostate Cancer		
			Gro.	Pro.	Met.
Andrographolide	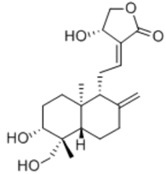	Diterpenoid lactone	+		
Evodiamine	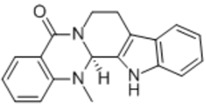	Alkaloid	+		
Guttiferone F	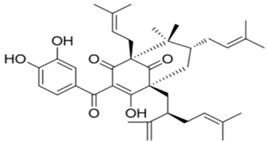	Prenylated benzophenone derivative	+		
Honokiol	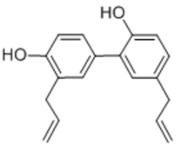	Aromatic phenol	+		
Isorhapontigenin	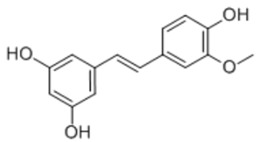	phytopolyphenol	+		
Peperotetraphin	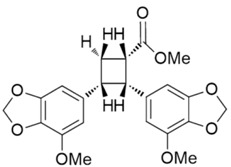	Norlignan	+		
Quercetin	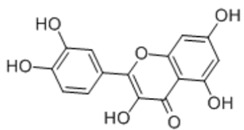	Flavone	+		
Tetrandrine	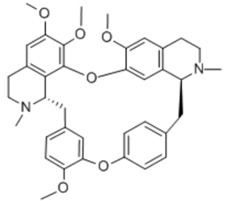	Alkaloid	+		+
Triptolide	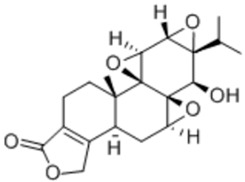	Heterocycle	+	+	
Vitexicarpin	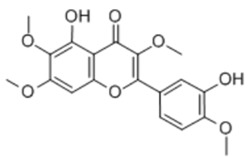	Flavone	+		
Arsenic sulphide	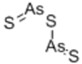	Metal chalcogenide		+	
Baicalin	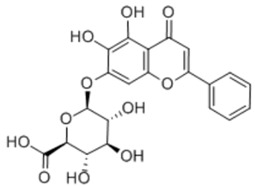	Flavone glycoside		+	
Celastrol	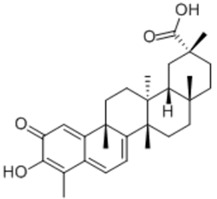	Pentacyclic triterpenoid		+	+
Gypensapogenin H	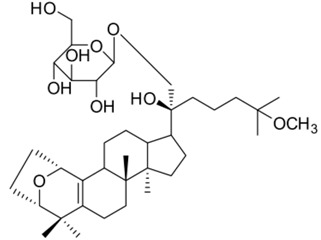	Triterpene		+	
Scoparone	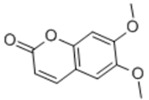	coumarin		+	
Curcumin	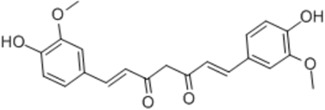	Diphenyl heptyl hydrocarbon			+
dihydroisotanshinone I	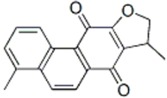	Phenanthraquinone			+
D-pinitol	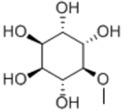	Alcohol			+
Shikonin	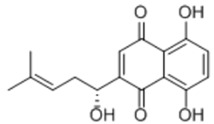	Anthraquinone			+

Note: “Gro” = Growth, “Pro” = Proliferation, ”Met” = Metastasis, “+” = Has effect on it.

**Table 3:** Activity of extracts and compounds isolated from Chinese medicines against prostate cancer cells in vitro.

Cell Type	Androgen-Dependent Human Prostate Cancer Cell Lines		Androgen-Independent Human Prostate Cancer Cell Lines				HPV-18-Transfected Human Prostate Cancer Cell Line	Mouse Transgenic Prostate Cancer Cell Lines		Mouse Prostate Cancer Cell Lines	
Name	LNCaP	22Rv1	PC3	PC-3M	DU145	C4-2B	CA-HPV-10	TRAMP-C1	TRAMP-C2	RM-1	Myc-CaP
*Ganoderma lucidum* (Leyss. ex Fr.) Karst.			+								
*Litchi chinensis* Sonn.			+		+	+				+	
*Saussurea lappa* Clarke					+				+		
*Scutellaria baicalensis* Georgi	+		+								
*Scutellaria barbata* D. Don	+							+			
*Tripterygium wilfordii* Hook F.			+		+						
*Wedelia chinensis* (Osbeck) Merr.	+	+	+								
Andrographolide			+		+						
Evodiamine	+										
Guttiferone F	+		+								
Honokiol	+		+								+
Isorhapontigenin	+	+									
Peperotetraphin			+								
Quercetin	+		+		+						
Tetrandrine			+		+						
Triptolide	+			+						+	
Vitexicarpin			+								
Arsenic sulphide			+		+						
Baicalin	+		+		+		+				
Celastrol			+								
Gypensapogenin H		+			+						
Scoparone					+						
Curcumin	+		+		+						
dihydroisotanshinone I		+	+		+						
D-pinitol			+		+						
Shikonin			+		+						

Note: “+” = has inhibitory effect on prostate cancer cell lines.
